# Clustering and curation of electropherograms: an efficient method for analyzing large cohorts of capillary electrophoresis glycomic profiles for bioprocessing operations

**DOI:** 10.3762/bjoc.16.176

**Published:** 2020-08-27

**Authors:** Ian Walsh, Matthew S F Choo, Sim Lyn Chiin, Amelia Mak, Shi Jie Tay, Pauline M Rudd, Yang Yuansheng, Andre Choo, Ho Ying Swan, Terry Nguyen-Khuong

**Affiliations:** 1 Analytics Group, Bioprocessing Technology Institute - Agency for Science Technology and Research. Singapore 138668; 2 University College Dublin, Belfield, Dublin, Ireland; 3 Animal Cell Technology Group, Bioprocessing Technology Institute, Agency for Science Technology and Research, Singapore 138668; 4 Stem Cells 1 Group, Bioprocessing Technology Institute - Agency for Science Technology and Research, Singapore 138668; 5 Department of Biomedical Engineering, Faculty of Engineering, National University of Singapore (NUS), Singapore 117575

**Keywords:** capillary electrophoresis, clustering, data analysis, electropherogram, glycosylation, monoclonal antibodies, peak picking, process development

## Abstract

The accurate assessment of antibody glycosylation during bioprocessing requires the high-throughput generation of large amounts of glycomics data. This allows bioprocess engineers to identify critical process parameters that control the glycosylation critical quality attributes. The advances made in protocols for capillary electrophoresis-laser-induced fluorescence (CE-LIF) measurements of antibody N-glycans have increased the potential for generating large datasets of N-glycosylation values for assessment. With large cohorts of CE-LIF data, peak picking and peak area calculations still remain a problem for fast and accurate quantitation, despite the presence of internal and external standards to reduce misalignment for the qualitative analysis. The peak picking and area calculation problems are often due to fluctuations introduced by varying process conditions resulting in heterogeneous peak shapes. Additionally, peaks with co-eluting glycans can produce peaks of a non-Gaussian nature in some process conditions and not in others. Here, we describe an approach to quantitatively and qualitatively curate large cohort CE-LIF glycomics data. For glycan identification, a previously reported method based on internal triple standards is used. For determining the glycan relative quantities our method uses a clustering algorithm to ‘divide and conquer’ highly heterogeneous electropherograms into similar groups, making it easier to define peaks manually. Open-source software is then used to determine peak areas of the manually defined peaks. We successfully applied this semi-automated method to a dataset (containing 391 glycoprofiles) of monoclonal antibody biosimilars from a bioreactor optimization study. The key advantage of this computational approach is that all runs can be analyzed simultaneously with high accuracy in glycan identification and quantitation and there is no theoretical limit to the scale of this method.

## Introduction

Glycosylation is important for the efficacy and function of a majority of the most dominant biologic drugs currently on the global market. In the case of antibody-based biotherapeutics, the absence of fucosylation or increase in galactosylation is needed for either antibody dependent cell cytotoxicity [1–2] or complement-dependent cytotoxicity [3–4], respectively, whilst additionally, antibody mannosylation is important for clearance [5]. For these reasons, glycosylation is a critical quality attribute (CQA) of most biologics. This necessitates control of glycosylation processing during a drug process development stage to ultimately relay a consistent glycosylation of the biologic product during manufacturing [6]. This is difficult because glycosylation during fermentation occurs with a high degree of heterogeneity and is influenced by several factors including the host expression system and process parameters such as temperature shifts, pH, and the type of basal/feed media [7]. To understand how these environmental factors impact the glycosylation of a biologic, analytical methods are needed to assess how glycans behave under these diverse conditions. During this process development of antibody-based drugs, the N-glycosylation of an antibody can deviate from their expected glycomic profiles as a result of fluctuations in culture conditions and operating parameters. Therefore, to assess antibody glycosylation accurately, high-throughput analysis of hundreds to thousands of profiles is required for the identification of critical process parameters that control the glycosylation CQAs [8].

For complete bioprocessing analysis, favorable glyco-analytical methods need to convey a qualitative description of the glycans, their relative abundance, and most importantly be high-throughput in terms of quantity, comprehensiveness, and speed of data generation. Capillary electrophoresis-laser-induced fluorescence (CE-LIF) is a glycomic analytical technology that has been adapted for automated and high-throughput analysis [9]. In CE-LIF, released and fluorescently labelled glycans migrate over a capillary and are identified by comparison to the standardized migration time with external or internal oligosaccharide standards. In order to achieve standardized migration time in a high-throughput manner, migration time is generally calculated by correlation with internal standards that bracket the time of elution of the glycans of interest [10]. This process is used to calculate a glucose unit (GU) which helps to align the datasets so that the GU of each glycan can be used to identify the glycan through available GU-based glycan databases [11–13]. The technique is suitable for the assessment of glycosimilarity of biologics [14] and most importantly has potential for analyzing large cohort studies to assess the aforementioned process parameters and their correlations with antibody glycosylation [7]. GU databases and software (among others) are discussed in a recent review [15].

A long standing problem associated with the analysis of large sets of electrophoretic data generated during bioprocessing is inevitably the drift of the peak migration time and area under the curve pertaining to glycan structures. This can be caused by a combination of sample complexity, temperature, pH, day of analysis, and other physicochemical fluctuations during the operation of the analysis. Although GU calculation can help solve this for the qualitative analysis, there is still difficulty automating peak picking due to small peaks and peaks that can lose their “Gaussian-ness” when multiple peaks migrate close together. This is especially true for large sets of diverse CE electropherograms collected over days or months under varied conditions. Consequently, they are often processed with automated software using different parameter settings for each electropherogram (or groups of similar electropherograms) requiring substantial human intervention to check correctness of the automated picked peaks and tuning parameters. This level of human manual data analysis is impractical when dealing with thousands of samples.

Here, we describe a computational solution for the identification and quantitation of glycans in a large glycomics CE dataset generated during process development of an anti-HER-2 antibody. The method is a semi-automated approach and improved accurate glycan assignments and quantitation compared to other tested fully automated software. Briefly, the method performs clustering analysis of glycomic electropherograms to group them into manageable clusters, followed by subsequent quantitation after semi-automated curation using the open source software HappyTools [16]. The clustering and migration time calibration in HappyTools allows for easy manual peak picking (spending 1 to 3 hours) before quantitation begins. After peaks are defined, large sets of electropherograms can be processed expediently and efficiently without any further need for human intervention either pre or post-quantitation. To the best of our knowledge, we are the first to apply this computational approach to a large set of CE-LIF glycomic data. The result of this new method is that large cohorts (thousands) of bioreactor runs can be analyzed at once with high accuracy in quantitation and glycan identification. We demonstrate this approach through the high-throughput qualitative and quantitation of CE-LIF glycomic data, displaying glycan trends that exist in eleven in-house bioreactor culture conditions. Most importantly we show that the quantitation is consistent with respect to other software. The key advantage of this computational approach is that all runs can be analyzed simultaneously with high accuracy in glycan identification and quantitation and there is no theoretical limit to the scale of samples that can be processed using this method.

## Results and Discussion

### Anti-HER-2 cultures

A comparison of a large set of glycosylation profiles derived from the bioprocessing and harvesting of Anti-HER-2 antibodies every day across 11 different culturing conditions. Specifically, Anti-HER-2 antibodies were harvested from 3 technical replicates for biological replicate across 12 days and 11 different culturing conditions (Supporting Information File 1, Table S1). Five of the replicates failed due to sampling errors, leaving a total of 391 electropherograms to identify and quantitate glycans. The N-glycans were enzymatically removed, fluorescently labelled with aminopyrene trisulfonate (APTS), and analyzed by capillary electrophoresis. The N-glycans were separated using a 5 minute separation across a 30 cm capillary. N-Glycan peaks in the electropherograms were annotated for all 391 electropherograms separately demonstrating that varying culture conditions resulted in significant differences in the electropherograms, i.e., certain glycan peaks became absent or present depending on the conditions and day of culture.

### Problems with automated identification and quantitation of glycans using Gaussian approximations

**Figure 1:**
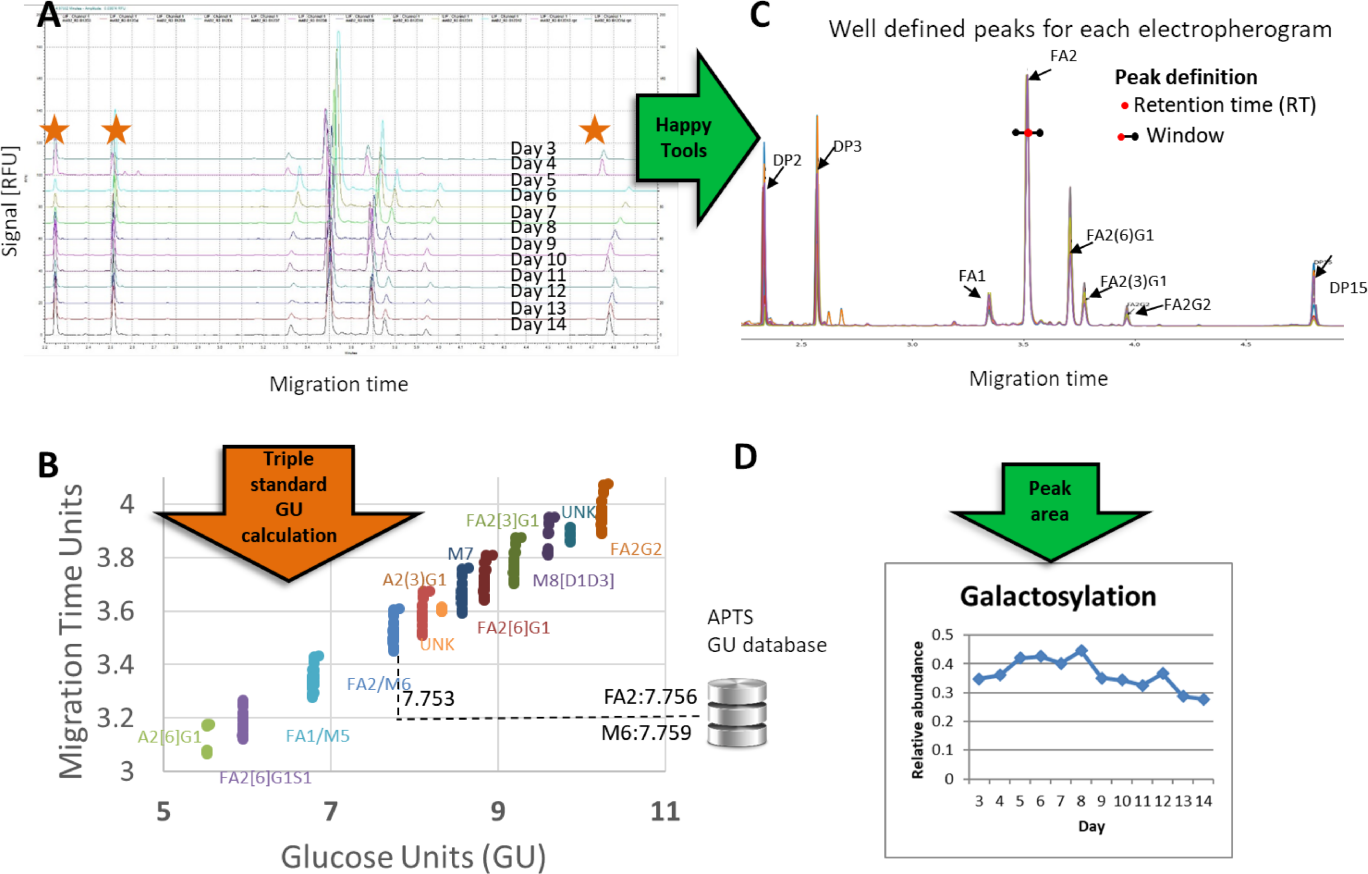
A single bioreactor run with defined culture conditions for twelve days. (A to B) batch GU calculation using the triple standard approach. The orange star marks the three bracketing standards. (B) Dotplot of the GU value vs. migration time. (A to C to D) HappyTools software allowed easier quantitation since all peaks can be aligned/calibrated and all peaks start and end migration times can be defined before quantitation begins using HappyTools.

Several approaches were examined to compare glycan identification and relative quantities between electropherograms for one set of results. This single set consisted of one bioreactor condition containing 12 days of CE-LIF electropherograms with 3 technical replicates (12 × 3 = 36 electropherograms). Figure 1 shows the approach we found to be optimal for this batch. The approach used to identify glycans was based on a triple standard GU calculation [10–11] and database matching whilst the quantitation used HappyTools calibration and area calculation [14]. The GU calculation involved standardizing the migration time of the peaks by generating a ‘virtual’ glucose unit (GU) ladder calculated using the migration time of the 3 oligosaccharide standards that were separated with each sample. The migration times of glycan peaks were then translated to a calculated GU that made it easy to compare peaks and identify glycans across electropherograms (Figure 1B). Despite major misalignment of migration time and bracketing standards in the electropherograms, the variation of GU values for glycan peaks generally were within a very small range (Figure 1A and 1B) allowing for consistent database matching against a GU CE database (APTS fluorescent labelled) [10].

HappyTools was used to calibrate the migration times of all the electrophoretic peaks (Figure 1C), define peak boundaries, and quantitate the glycans. Migration time calibration involved aligning peaks so that each glycan peak fell under the same migration time (Figure 1C). After alignment/calibration the peaks were easy to define manually and thus quantitation could be achieved using defined peak windows, migration time positions, and HappyTools peak area calculations (Figure 1C and 1D). Quantitation could be achieved with a simple user interface and HappyTools returned the glycan profile and quantitation results efficiently.

Unfortunately, the automated quantitation with HappyTools (Gaussian mode) and other software were hampered by complications in peak picking and peak area calculations of non-Gaussian peaks (see next section). This required significant time (2–3 days) to manually inspect and correct the quantitative values of the peaks in the subset of samples. The simplified approach shown in Figure 1 although useful for electropherograms with homogenous peaks would not be practical given the scale of sample numbers and heterogeneous nature of the samples we needed to investigate. Further investigation and alternative approaches were needed to facilitate better peak picking and quantitation with electropherograms that were composed of heterogeneous peak shapes in our glycan analysis.

### Peak detection and quantitation of non-Gaussian peaks using Riemann approximation

**Figure 2:**
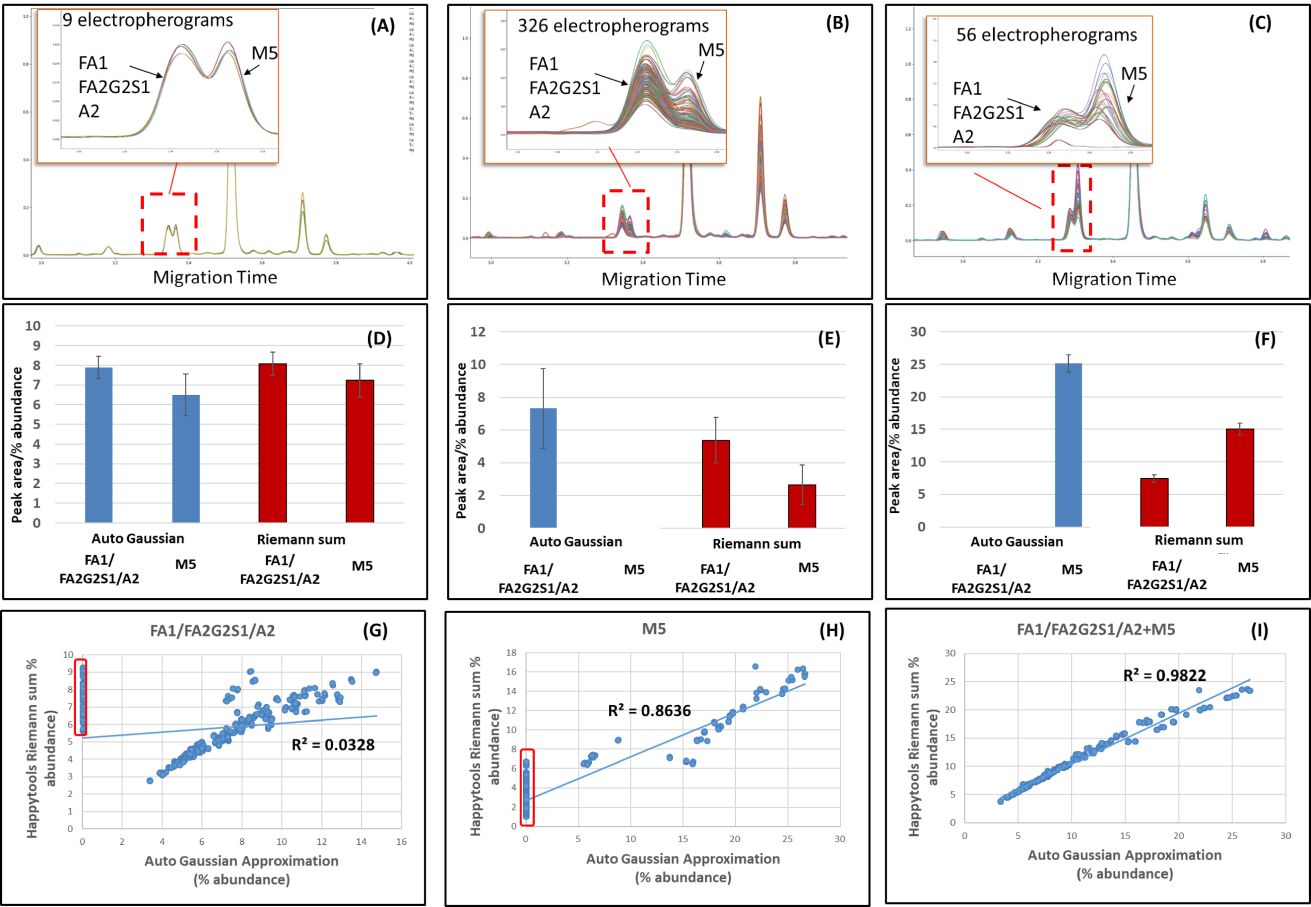
Problems when integrating poorly resolved peaks using FA1/FA2G2S1/A2 and M5 peaks as an example. (A) FA1/FA2G2S1/A2 and M5 had similar peak areas. (B) FA1/FA2G2S1/A2 had a greater peak area than M5. (C) FA1/FA2G2S1/A2 had less peak area than M5. (D) Average peak area and standard deviation (error bars) for 9 electropherograms in A. (E) Average peak area and standard deviation (error bars) for 326 electropherograms in B. (F) Average peak area and standard deviation (error bars) for 56 electropherograms in C. (G). Correlations between 32 Karat and HappyTools/Riemann sums for FA1/FA2G2S1/A2 only (red box peak not picked by 32 Karat), (H) M5 peaks only (red box peak not picked by 32 Karat, and (I) when FA1/FA2G2S1/A2 and M5 peak areas were combined there was excellent correlation between both approaches suggesting 32 Karat integrates the two peaks as a whole.

Quantitation of sets of electropherograms that were similar (i.e., technical replicates or biological replicates with the similar operating conditions) was feasible by manually tuning the parameters in software such as 32 Karat (Sciex) [17] (results not shown). However, when there was large heterogeneity in the CE-LIF electropherograms, as would be the case in a bioprocessing operation, a single set of tuned parameters failed to detect peaks and therefore quantitate them. On our dataset, the 32 Karat software needed tuning of parameters for multiple clusters of similar electropherograms; this job was laborious and thus motivated the implementation of our computational approach. The main reason quantitation was complicated by automated data analysis methods, whether using 32 Karat software [17] or HappyTools quantitation functions, was because of Gaussian peak approximation [16]. Using 32 Karat with a single set of default parameters there were difficulties with consistently peak picking closely eluting peaks and this led to inconsistent peak quantitation. Figure 2 shows two peaks that had similar migration time containing glycans FA1/FA2G2S1/A2 and M5 (identification results shown later). Using default settings, the 32 Karat software gave a peak area in one of three ways: for both (Figure 2A), only FA1/FA2G2S1/A2 (Figure 2B), or only M5 (Figure 2C). Similar anomalies in the FA1/FA2G2S1/A2 and M5 peak quantitation were also found when we attempted to fit the peaks using the Gaussian functions in HappyTools (results not shown). Peak quantitation was improved once we switched to non-Gaussian area calculation in HappyTools that used a Riemann sum between manually determined start and end migration times. The Riemann sum setting was recommended previously [16] for the quantitation of asymmetric, non-Gaussian peaks. In Figure 2, the improvement is shown for the FA1/FA2G2S1/A2 and M5 peaks where their relative sizes on the electropherogram compared well with the Riemann sum peak area calculations, i.e, almost equal areas (Figure 2A vs 2D), FA1/FA2G2S1/A2>M5 (Figure 2B vs 2E), and FA1/FA2G2S1/A2<M5 (Figure 2C vs 2F). Further, peak area consistency was achieved between 32 Karat and the HappyTools/Riemann sum approach when FA1/FA2G2S1/A2 and M5 peaks were combined (Figure 2G and 2H vs 2I). Thus, both approaches had consistent peak area calculations when considering the two peaks as one. However, the HappyTools/Riemann sum approach was advantageous because it allowed for a separation of the two distinct peaks thus giving a finer level of glycan detail.

### Problems aligning and comparing the large cohort data with Gaussian modelling of electrophoretic data

**Figure 3:**
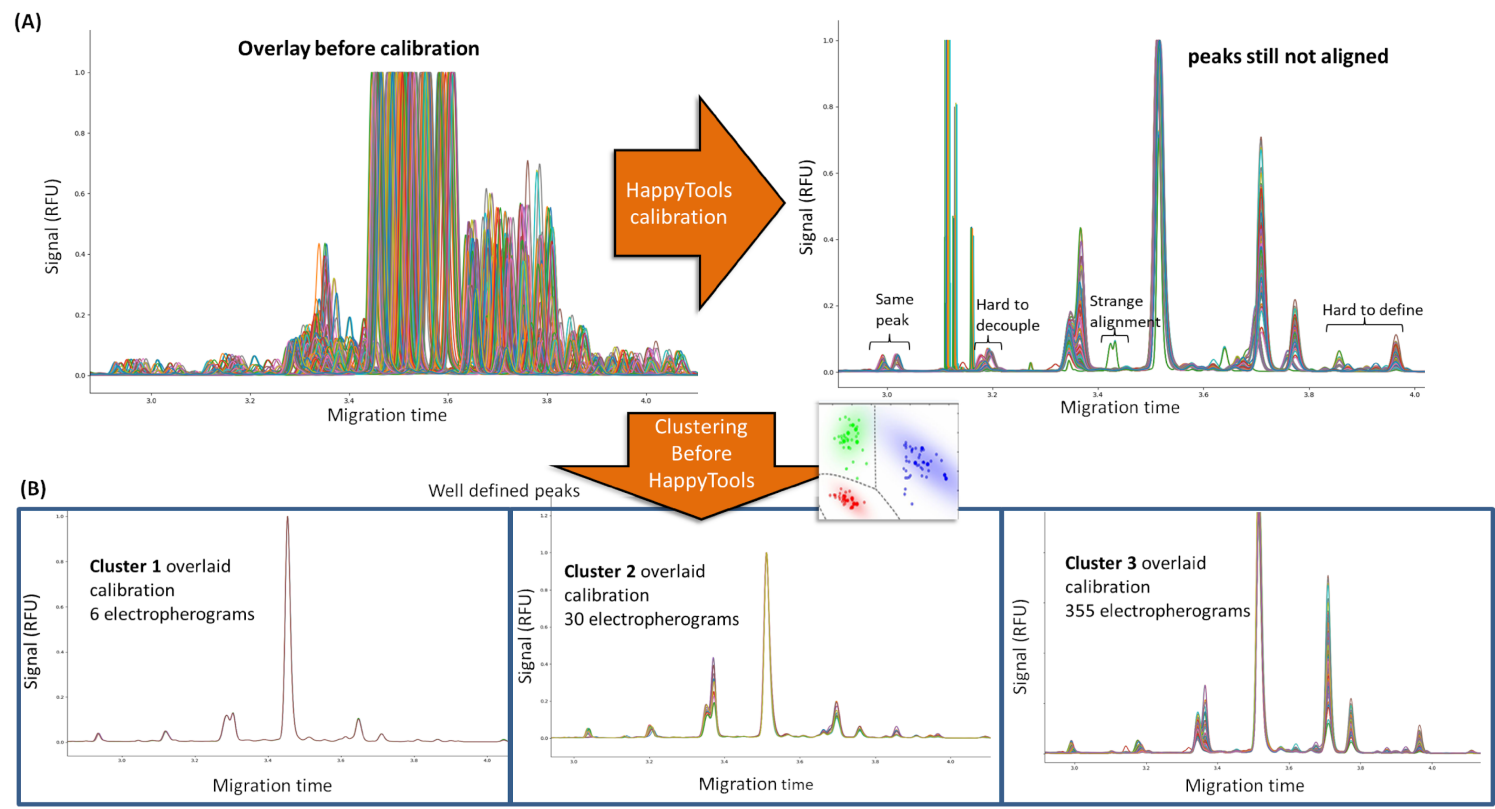
The clustering function allowed grouping of similar electropherograms and therefore clean the HappyTools calibration output. (A) HappyTools calibration of all 391 electropherograms was largely misaligned. This made peaks not well aligned and made it difficult to define the peak positions for quantitation. (B) The clustering grouped the HappyTools calibration into three clusters of similar electropherograms. Each cluster had well defined peaks that were manually determined.

HappyTools calibration and quantitation worked well with the single bioreactor condition shown in Figure 1A–C because the electropherograms were similar. Upon expanding the same analysis workflow across all 391 electropherograms, inconsistent calibration was observed for the different electropherograms, resulting in peaks that were hard to define (Figure 3A). The reason for this difficulty in defining peaks was because of peak misalignment caused by the heterogeneous nature that resulted from fluctuations in day-to-day electrophoretic operating conditions such as temperature, voltage changes, etc. For some electropherograms, differences were the result of new glycan peaks attributed to the biological variations introduced via multivariate culturing conditions. We therefore implemented a clustering algorithm that allowed us to group electropherograms before applying HappyTools calibration (Figure 3B).

### Clustering and manual peak picking efficiently and comprehensively quantitate large cohorts of glyco-profiles

The electropherograms were grouped using unsupervised clustering and the peak intensity as input variables. From our analysis, the 391 electropherograms were clustered into three distinct groups (Figure 3B) of electropherograms. Visualization by overlaying the electropherograms in each cluster (Figure 3B) showed that it was easier to define user-generated peak migration times and delta-windows (Supporting Information File 1, Table S2). The clustering simply facilitated manual peak picking thus avoiding the pitfalls associated with automated peak picking. The manually determined data in Supporting Information File 1, Table S3 was transferred to HappyTools quantitation Riemann peak area functions via its analysis file. Therefore, once we defined the peaks manually our clustering + HappyTools computational approach could quantitate similar groups of electropherograms separately on a large scale. In total, Supporting Information File 1, Table S3 shows there were 17 peaks manually identified that required glycan annotations and quantitation.

**Figure 4:**
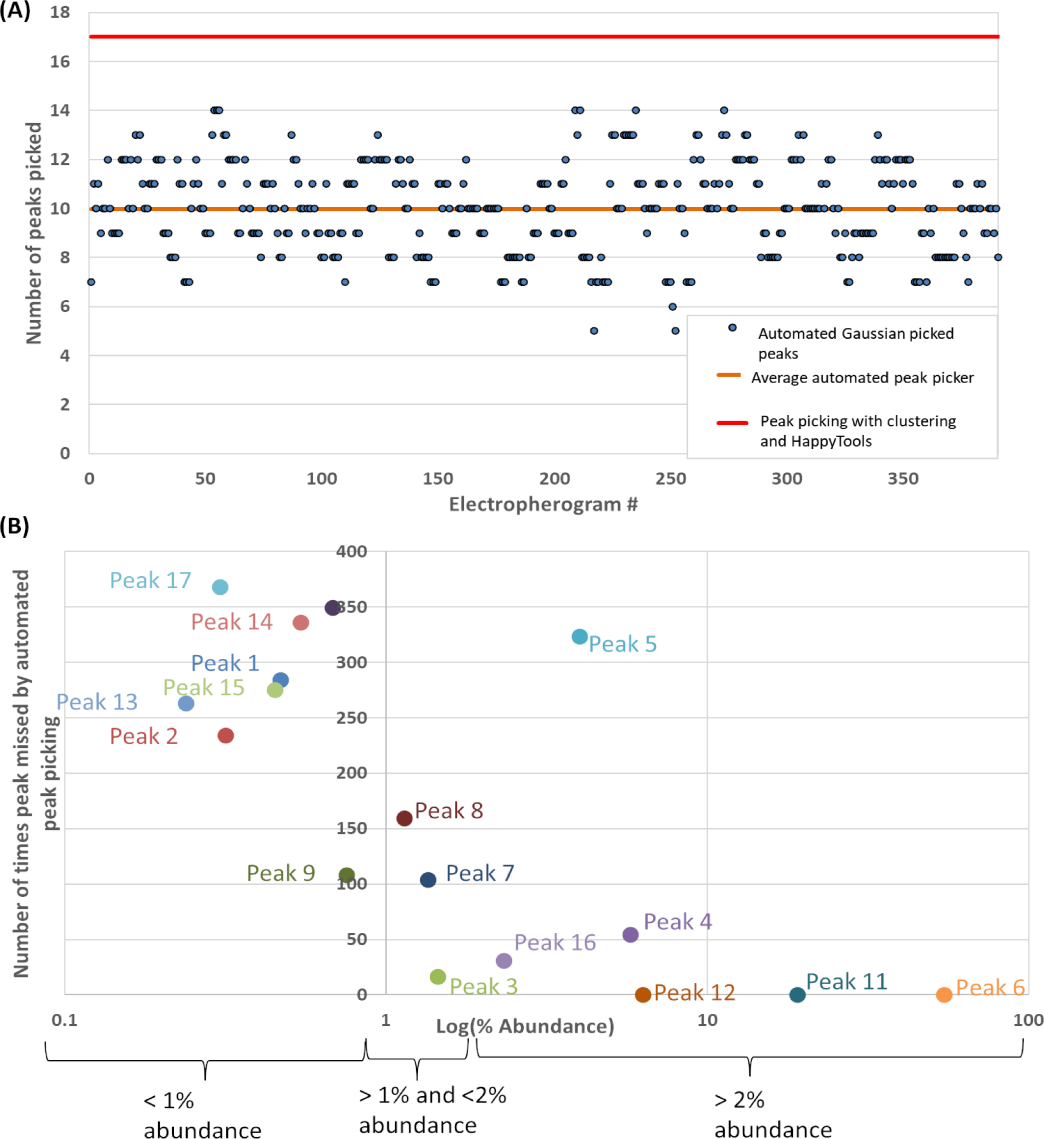
Comparison of the performance of the automated peak picking and semi-automated clustering and HappyTools quantitation for the 391 electropherograms. (A) Automated quantitation using Gaussian approximation approach picked on average 10 peaks (min = 5, max = 14) while our HappyTools + Clustering approach constantly picked 17 peaks. (B) Number of times automated Gaussian peak picking missed one of the 17 peaks listed in Supporting Information File 1, Table S3 as a function of % abundance.

The semi-automated approach of clustering electropherograms combined with manual peak curation and HappyTools consistently outperformed automated approaches: 32 Karat (Sciex) and HappyTools automated functionality. On close inspection, it was noted that peak integration under a Gaussian approximation would yield a high variation in the number of picked peaks per electropherogram (Figure 4A). The number of peaks picked using the automated approaches was on average 7 peaks lower than our clustering, manual peak curation, and quantitation using HappyTools (Figure 4B). Furthermore, for low abundant peaks and peaks with close migration times, automated peak quantitation randomly misses out peaks which were discovered using clustering and manual curation (Figure 4B). These low abundant peaks accounted for a significant share of the glycan peak abundance. Peaks that constituted <1% abundance account for 3.8% of the total average abundance. Peaks that constituted 1–2% abundance accounted for 4.0% of the total average abundance and peaks >2% accounted for 92.2% of the average total abundance. If peaks <1% are not considered it might seem insignificant but it affects the relative abundance of other peaks to an extent of 3.8% in total.

Supporting Information File 1, Figure S1 shows the correlation between clustering + HappyTools and the automated software quantitative strategies. The high correlations in Supporting Information File 1, Figure S1 for the major peaks show that the quantitative calculations of both approaches were similar. However, as mentioned previously we found that the quantitation algorithms provided by the automated software required a substantial amount of human intervention (2 to 3 days approximately). The manual checking was needed because of missing peaks (Supporting Information File 1, Figure S1; red rectangles, Figure 4) and erroneous peak area calculation for close peaks (Figure 2). The user would have to identify exactly which samples out of hundreds or thousands of samples were incorrectly determined, introducing human error and slowness back into the automated process. On the other hand, our clustering + HappyTools the quantitative approach was quick, taking only 2 minutes on a standard personal computer for all 391 electropherograms. However, there was the need to manually define the peaks in Supporting Information File 1, Table S3 which required 1 to 2 hours of examining the electropherogram overlays in Figure 3B. At this stage we had optimized our quantitation protocol but prior to applying the quantitation algorithm, we needed to identify what glycans were eluted at each peak.

### Glycan identification for all 11 bioreactor runs

**Figure 5:**
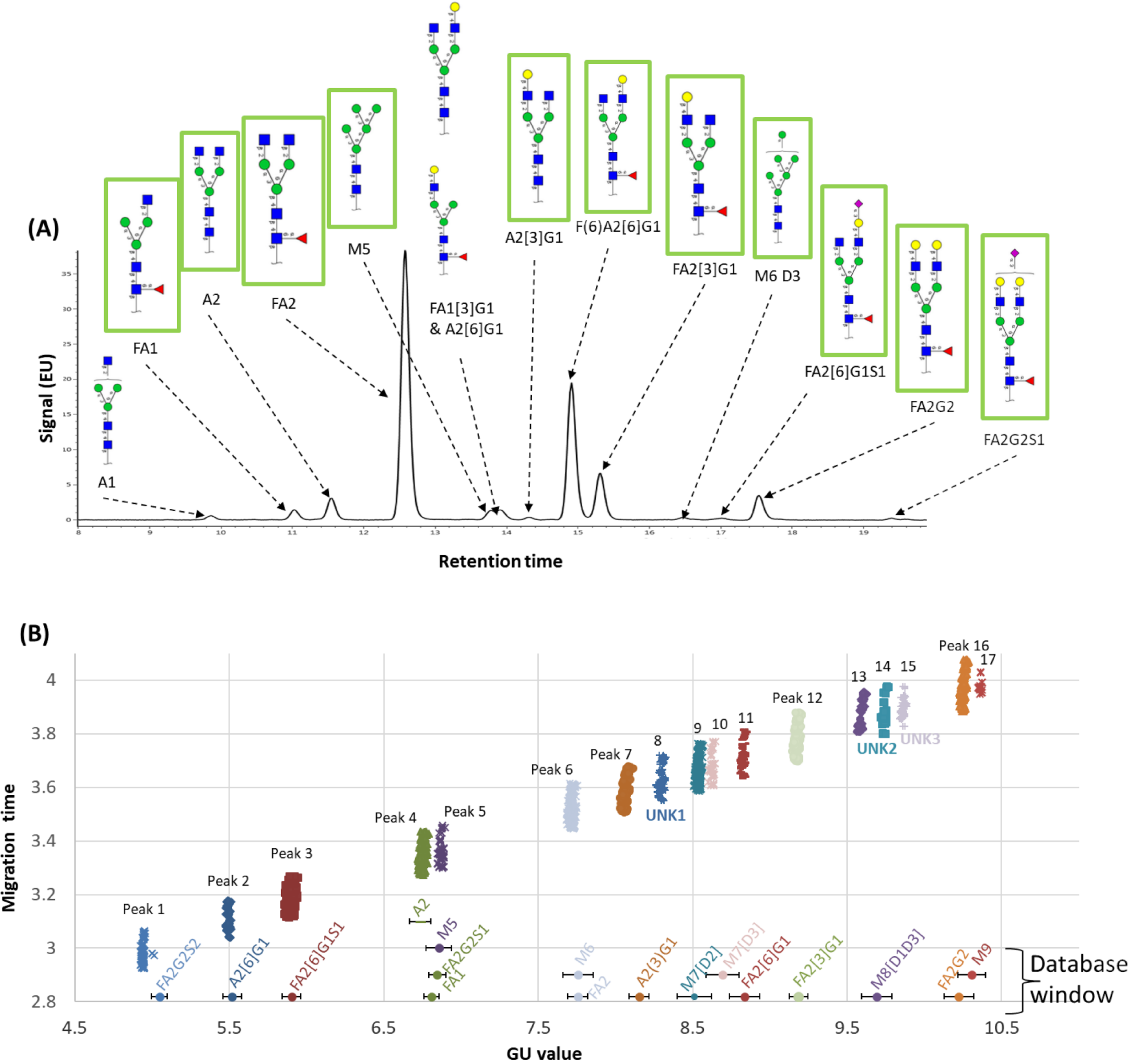
Glycans identified in anti-HER-2 samples using UPLC-MS and CE. (A) the UPLC chromatogram confirmed the 14 glycans using GU and mass. Green boxed glycans were also identified in CE. (B) Glucose units vs. migration time for all 391 CE electropherograms. Database matched glycans are shown in Oxford linear notation [19]. The CE APTS database hits are marked with a circle and a corresponding error bar showing the GU tolerance. All glycans with core fucose were α-1→6 linkage, galactose were β-1→4 linkage and all sialic acid linkages were α-2→3 linkage. All glycans are drawn in SNFG notation [20].

Glycan identifications to all peaks in all 391 electropherograms were confirmed using evidence from two orthogonal approaches, UPLC-MS (RFMS fluorescent label) analysis and a CE-LIF analysis (APTS label). The UPLC-MS approach was used to characterize a commercial Anti-HER-2 reference standard (Section 4.3.4 from mentors) using the UNIFI software and GU/mass database (RFMS labelled) provided by Waters Corp [18]. Figure 5A shows that 14 glycans were identified in 13 UPLC peaks using UPLC-MS glycomics analysis. Figure 5B shows that 17 glycans were identified for 14 peaks using CE. Out of the 17 glycans, there were 11 also confirmed by UPLC-MS (Figure 5A green boxes; Supporting Information File 1, Table S4 green column headers) thus increasing assignment confidence for those. Out of the 17 peaks in Supporting Information File 1, Table S3, 14 could be assigned glycan structures (Figure 5B) using GU database matching. Figure 5A shows that 3 UPLC-MS identified glycans, A1, FA1[3]G1, or A2[6]G1, were not found in the CE-LIF analysis. The reason for lack of A1 and FA1[3]G1 annotation in the CE-LIF was likely because they were not in the APTS GU database. Therefore, glycans A1 and FA1[3]G1 could be any one of the 3 unidentified glycans in the CE-LIF (Figure 5B marked UNK1, UNK2 and UNK3) and further investigation is needed. Glycan peak A2[6]G1 was in the APTS database with a GU of 8.153 and it did not match any of our peaks in Figure 5B. The fact that there were 17 glycans identified in the CE-LIF and 14 in UPLC-MS seems counter-intuitive but it can be explained by the vast degree of variation in our bioreactor conditions while the anti-HER-2 innovator was produced from a single harvested condition.

Using the UPLC-FLR-MS quantitation we could estimate the area under the curve contribution for each glycan in the co-eluting peaks 4 and 6. In UPLC-FLR-MS, FA2G2S1 was very minor (0.21%), while FA1 and A2 had an abundance of 1.8% and 3.9%, respectively. This suggests that A2 is the major component of peak 4 followed by FA1. Similarly, for FA2/M6 UPLC-MS quantitation showed FA2 with 49.3% and M6 with 0.32% abundance, respectively, suggesting the M6 component was relatively smaller for peak 6 quantitation. The ability to estimate the relative contributions of glycans in a co-eluting peak was another advantage of combining our CE analysis with UPLC-MS characterization.

### Peak quantitation for all 11 bioreactor runs and all 17 peaks

**Figure 6:**
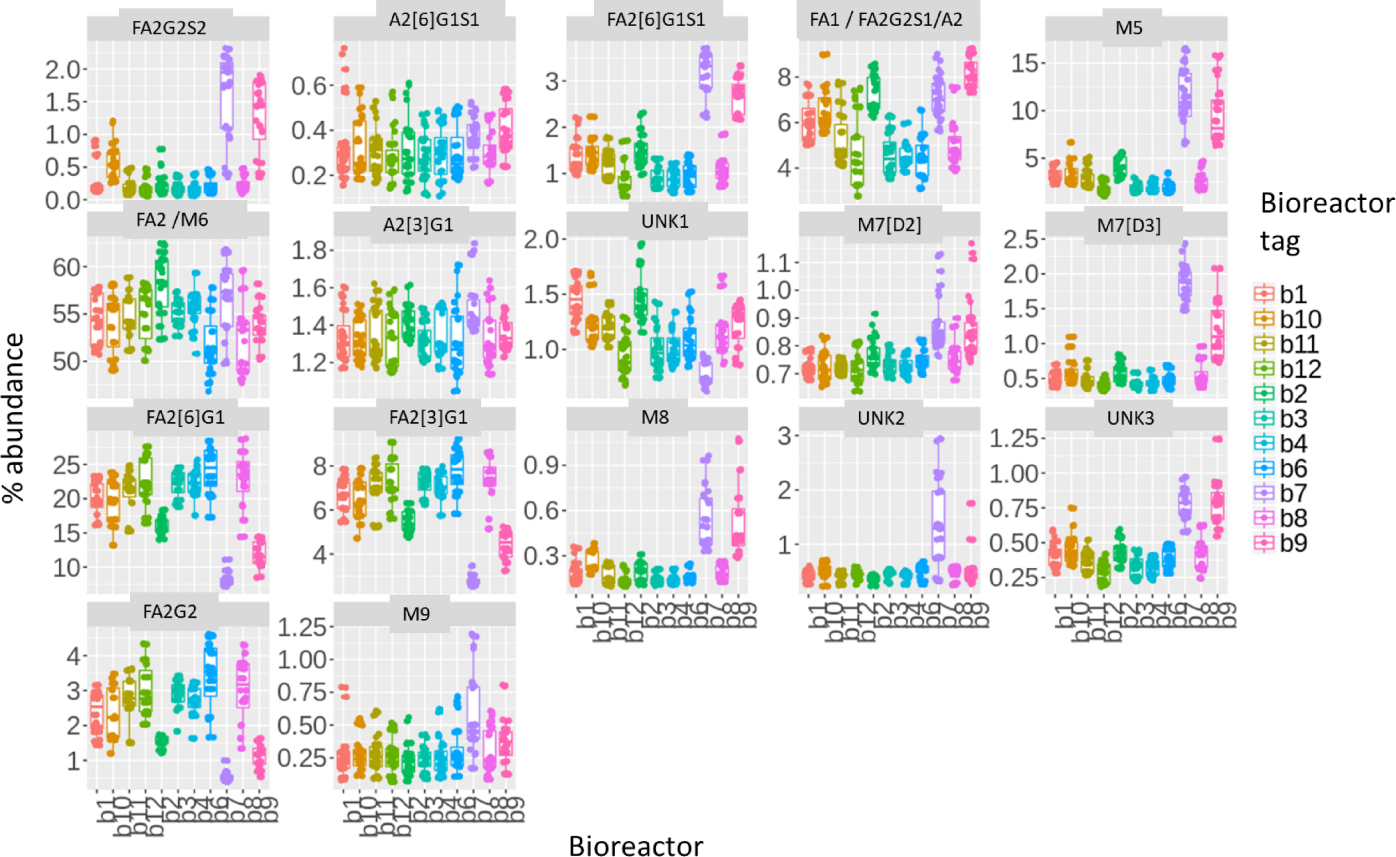
Boxplots showing the quantitation of the 11 different bioreactor conditions. The boxplots show the peak area distribution (expressed as % relative abundance) for each of the 11 bioreactor conditions. Points are averaged relative abundances for three replicates.

Upon successful calibration and annotation of the 391 samples separated by CE-LIF, and analysis using clustering + HappyTools, we were able to compare the overall glycosylation profiles that resulted from the different bioprocessing operating conditions. Figure 6 shows the final quantitation for all 17 peaks in Supporting Information File 1, Table S3 and all glycans using our proposed computational approach. The final relative abundances were an averaged percentage value from three technical replicates resulting in 132 observed glycan profiles. The variability between technical replicates was low as shown by their standard deviations in Supporting Information File 2. Conversely, the variability between bioreactor conditions was high and a number of other interesting observations can be concluded from the quantitation including: a) core fucose sialic acid based glycans had increased abundances in bioreactor condition b7, b8, and b9, b) mannosylation was increased in b7 and b9, c) neutral fucosylation decreased for b7 and b9. However, FA1 did not follow this trend perhaps because it was co-eluting with A2 and FA2G2S1, d) neutral galactosylation decreased for b7 and b9, e) fucosylated and galactosylated glycans FA2G2, FA2[6]G1, and FA2[3]G1 decreased for b7, b9 and b2. Conversely, the fucose agalactosylation glycan FA2 was increased for the b2 condition suggesting a change in the galactose transferase activity in the b2 culture condition. Additionally, the unidentified peaks in the qualitative analysis (UNK1, UNK2, and UNK3) had very small impact.

Supporting Information File 1, Figure S2 shows the intercluster quantitation variation for clusters 1, 2, and 3. It shows different quantitation patterns for 7 abundant glycans (average > 1% abundance) in each cluster. Interestingly, cluster 1 and 2 (Figure 3) were mainly composed of two particular bioreactor conditions (b7 and b9; Figure 6) and contained higher levels of FA2, FA1, and M5 and lower levels of FA2[6]G1 and FA2[3]G1 (Supporting Information File 1, Figure S2). This suggested that the clustering was also useful to group electropherograms based on culture conditions and glycan abundances and could be an important characteristic of the approach as it could allow quick identification of bioreactor conditions important for some glycan types.

### Future work: process development of the glycans against various physicochemical parameters of the bioprocess

This work reports a computational pipeline we utilized for batch identification and quantitation of glycans in CE electropherograms from a large sample cohort. This approach will be subsequently utilized to evaluate the time-based glycosylation profiles of an antibody product (and therefore biologic quality) derived from bioreactors operating under varying culture conditions. The number of glycosylation profiles that can be processed has no theoretical upper limit. In this work, the computational approach was optimized on one bioreactor sample set and proved to be efficient and accurate. In future work we will apply this approach to substantially more bioreactor culture conditions thus producing massive amounts of data that could be used to optimize bioprocessing and biologic quality.

## Conclusion

Fluctuations of glycomic profiles are a result of environmental and biochemical pathways of the glycoprotein. Understanding these effects requires a high-throughput analytical technique whereby diverse glycomic profiles of the glycoprotein/s can be compared qualitatively and quantitatively. For capillary electrophoresis-based glycomics data processing, this is complicated by heterogenous glycan peaks which may not fit under a Gaussian approximation. As such, automated Gaussian fitting of these peaks will yield inaccurate representation of the glycans expressed in the complex system. This is particularly true for peaks with very close migration times.

We observed that for large sets of heterogeneous electropherograms current software for quantitation was inadequate (although software such as 32 Karat proved excellent for glycan identification). We therefore describe a method whereby we perform clustering analysis of large cohorts of glycomic CE-LIF electropherograms, breaking them down into smaller, more manageable groups of similar electropherograms, followed by using open-source software for quantitation. The computational approach can be adapted to any analytical technique that produces large amounts of heterogeneous profiles as it allows for easy manual peak picking before quantitation begins. After peaks are defined, large cohorts of profiles can be processed expediently and accurately without any further need for human intervention pre or post-quantitation. Thus, the approach is semi-automated, achieving the scale of automation while still maintaining the accuracy of manual assignment.

We used this technique to comprehensively and accurately characterize the effect of multivariate bioprocessing conditions upon the glycosylation profile of an anti-HER2 antibody product. We found that our clustering + Happytools method reduces 2–3 days of human intervention needed for the automated software down to 1–2 hours for first-time analyses, but down to minutes for repeated analyses. We envision that this approach may be widely applicable to large cohort glycomic studies, where the comparison of glyco-profiles is important to clinical studies, cellular biology, and glycobiology in general.

## Experimental

### Materials

Sodium phosphate (Merck) and glycine·HCl (Merck) were purchased from Merck. Tris-HCl and EX-CELL Advanced CHO Fed-batch medium were all purchased from Sigma Aldrich. ACN (Part no: A955-4, Fisher Scientific), PVDF syringe filter (Part no: SLGV013SL, Millex 0.22um PVDF 13 mm sterile syringe filter) were obtained from Merck Millipore (Ireland), whilst centrifugal filters, (Part no: UFC3096, Amicon Ultra Centrifugal Filters) was purchased from Merck Millipore, (USA). Protein A HP Spintrap (28-9031-32) was purchased from GE Healthcare, USA. FAST Glycan Kit (Part no: B94499PTO, SCIEX, USA). Ammonium formate (Part No. 186007081), RapiGest SF (Part No. 186001860), RapiFluor-MS Reagent Solution (Part No. 186008091), and ACQUITY UPLC^®^ Glycan BEH Amide Column were purchased from Waters Corp.

### Cell culturing of samples

CHO-K1 cells producing Adalimumab biosimilar were cultured in 14-day fed-batch cultures using Ambr250 bioreactors. The cells were thawed and passaged three times in 30 mL of EX-CELL Advanced CHO Fed-batch medium supplemented with 6 mM of glutamine and 250 nM of MTX in 50 mL tubespin cultures prior to bioreactor inoculation. Cells were inoculated into 200 mL of EX-CELL Advanced CHO Fed-batch medium supplemented with 6 mM of glutamine but without MTX at a viable cell density of 3 × 10^5^ cells/mL. The cultures were mixed using dual pitch blade impellers stirring at 300 rpm. Different bioreactor operating conditions were evaluated. Triplicate experiments were performed for all operating conditions under study. In terms of the feeding strategy, 10% of EX-CELL Advanced CHO Feed 1 (with glucose) were added to all cultures on days 3, 5, 7, 9, and 11. When the concentration of glucose dropped to below 2 g/L, a specified volume of 45% glucose stock was added into fed-batch cultures in order to achieve a final glucose concentration of 6 g/L. Glycosylation analysis was performed for samples obtained daily from days 3 to 14.

### Sample preparation of antibody N-glycans

**Protein A purification and buffer exchange:** The collected cell supernatant was filtered through a 0.22 µM PVDF syringe filter (Sterile Millex Filter, Merck Millipore, Ireland). Antibodies were then purified using protein A spin trap columns (Protein A HP Spintrap, GE Healthcare, USA). The columns were equilibrated and washed via centrifugation (100*g*, 30 s) with 20 mM sodium phosphate (pH 7.0). The sample was loaded to each column (maximum volume of 600 µL) and incubated end-over-end for 10 min. The column was washed with 20 mM sodium phosphate (pH 7.0) via centrifugation (100*g*, 30 s). The antibody samples were eluted from these columns with 0.1 M glycine·HCl (pH 2.7) and neutralized with 1 M Tris-HCl (pH 8.0). Samples were then buffer exchanged using 30 kDa Centrifugal filters, (Amicon Ultra Centrifugal Filters, Merck Millipore, USA) into dH_2_O and dried into 100 µg aliquots using a CentriVap benchtop vacuum concentrator (Labconco, USA).

**Free N-glycan labelling with APTS:** Free-N-glycans from purified antibodies were labelled with 8-aminopyrene-1,3,6-trisulfonic acid (APTS) using the FAST Glycan Kit (SCIEX, USA). Digestion, denaturing and labelling solutions were made according to the manufacturer’s instructions. This protocol was adapted to a 96-well PCR plate. Two hundred (200 µL) of magnetic beads were used per 100 µg of glycoprotein. The magnetic bead storage solution was removed using a plate magnet. Antibodies of 100 µg in 10 µL (10 µg/µL) aliquots were added to the beads. The samples were incubated for 8 min at 60 °C with the denaturing solution. Then, digestion solution was added, and the sample was incubated for 20 min at 60 °C. Acetonitrile was then added to the sample, and then placed on a magnetic plate to separate the beads from the supernatant. The supernatant was removed, labelling solution containing an internal standard (DP3) was added, and samples incubated at 60 °C for 20 min in the dark. After incubation, a cleanup solution and acetonitrile were added, followed by separation on a magnetic plate, and removal of supernatant. This cleaning step was repeated a further 2 times. The labelled glycans were eluted from the beads using 100 µL deionized water and placed on the plate magnet. Eluted labelled glycans were then stored at −21 °C in the dark until further analysis.

### Analysis of the released glycans using capillary electrophoresis

Capillary electrophoresis of the released and APTS-labelled antibody N-glycans was performed on a CESI8000 CE instrument (Sciex) equipped with a solid state laser-induced fluorescent detector (excitation 488 nm, emission 520 nm). Separations were made across a 20 cm effective length (30 cm total length), 50 µm i.d. uncoated bare fused capillary, HR-NCHO separation gel buffer (Sciex). The applied electric field strength was 1500 V/cm with the cathode at the injection side and the anode at the detection side (reversed polarity). Samples were electrokinetically injected using 1kV for 5 s. For migration time correction, a bracketing standard (BST) was co-injected with each sample. Samples were run in triplicate and a blank water injection without BST was run periodically throughout the analysis. 32Karat version 10.1 was used to control the instrument

### Analysis of Anti-HER-2 innovator released glycans using UPLC-MS

The supernatant was purified using Protein A HP SpinTrap (GE Healthcare). The purified glycoprotein obtained was buffer exchanged into water using a 10 kDa molecular weight cut-off filter (Merck Millipore) to eliminate any salts and nucleophiles that could interfere with the subsequent steps.

N-glycans were analyzed from the anti-HER-2 innovator monoclonal antibody using the RapiFluor-MS (RFMS) N-glycan kit (Waters Corp). Fifteen micrograms (15 µg) of glycoprotein was dried down and reconstituted in a digestion buffer (final concentration 0.01% RapiGest) and heated to 95 °C for 5 min to denature the glycoprotein. After the mixture was cooled to room temperature, 600 U of recombinant PNGase F (New England Biolabs) were added. The mixture was incubated at 55 °C for 10 min to enzymatically cleave the N-glycans from the protein and then cooled to room temperature. RapiFluor-MS Reagent Solution (0.07 mg/µL in anhydrous dimethylformamide (DMF, Waters Corp.) was added to the released glycans, and the labelling proceeded at room temperature for 5 min. The reaction mixture was diluted by adding acetonitrile (ACN; final concentration 89.5%) in preparation for HILIC SPE. Purification of the RFMS-labelled glycans was performed using a 96-well GlycoWorks HILIC μElution Plate (Waters Corp). The plate was initially equilibrated with sequential washes with dH_2_O and 85% ACN. The samples were loaded onto the wells and washed with 90% ACN/1% formic acid (FA). The glycans were eluted with GlycoWorks SPE Elution Buffer (Waters Corp). The eluted glycans were dried and reconstituted in a 22.5% (v/v) DMF, 25% (v/v) ACN. The glycans were analyzed via UPLC-MS using a H-Class UPLC equipped ACQUITY UPLC^®^ Glycan BEH Amide Column, (130 Å, 1.7 µm, 2.1 mm × 150 mm) (Waters Corp) which was coupled to a Xevo G2S QToF (Waters Corp). The flow rate was set at 0.4 mL/min and a linear gradient was used: 25–49% of buffer A (50 mM ammonium formate solution, pH 4.4) and buffer B (100% ACN) was run across 40 minutes, followed by a 3 min wash step using buffer A. The column was then equilibrated back to 25% buffer A. The labelled glycans were detected with an FLR detector (Ex 265 / Em 425 nm). The sample manager was set at 10 °C and the temperature of the column was kept at 60 °C throughout the analysis. Glycan masses were measured on the Xevo G2S QToF using sensitivity mode in positive mode. A mass range of *m*/*z* 400–2000 was used, with an acquisition speed of 1 Hz, and the mass spectrometry was set at the following conditions: 2.75 kV electrospray ionization capillary voltage, 15 V cone voltage, 120 °C ion source temperature, 300 °C desolvation temperature, 800 L/h desolvation gas flow. A lockspray [Glu1]-Fibrinopeptide B Standard (Waters Corp.) was also used throughout the run to maintain mass accuracy. Dextran ladder (Waters Corp.) was run to obtain a calibration curve with a cubic spline fit. The retention times were normalized using the calibration curve to glucose units (GU). The data obtained was processed and analyzed with the UNIFI Biopharmaceutical software platform (version 1.8).

### Data analytics

**Qualitative protocol:** Sciex 32Karat version 10.1 included a GU Value calculation component (FastGlycan) that was used to identify glycans in the acquired data. It is based on the triple standard approach previously described [10–11]. Glycans were matched to an GU-CE APTS database by finding the closest GU value in the database to the observed GU value. For UPLC-MS the data obtained was processed and analyzed with the UNIFI Biopharmaceutical software platform (version 1.8) where glycans were matched using the internal UNIFI RFMS GU-mass database and corresponding functions.

**Quantitative protocol:** Our quantitative approach consists of two software components: HappyTools previously described [16] and our in-house clustering algorithm. HappyTools was first used to calibrate/align the electropherograms. HappyTools performed calibration by examining user defined calibrant peak list consisting of: the third bracketing standard DP15, consistently highly abundant Anti-HER-2 glycan peaks such FA2, FA1 and FA2G2. This gave a good spread of calibration peaks across the electropherograms. For details on the calibration algorithm see [16]. The calibrated electropherograms were then clustered. The clustering algorithm was implemented in-house using the SciPy python package. The clustering step consists of hierarchical clustering using a single linkage algorithm and forms flat clusters using the inconsistency method with a cut-off threshold of 0.7, which was determined as achieving the same discrimination as manual classification on some test electropherograms. The data points presented to the clustering algorithm were an array of continuous signal intensities between migration time 2.9 and 4.1 (i.e., the Anti-HER-2 peaks). The pairwise similarity between any two electropherograms was calculated using Euclidean distance metric. The clustering algorithm is presented in Supporting Information File 1, Figure S3. After clustering, each cluster contained N electropherograms and each peak's central migration time (CRT) and window (ΔW) were defined by visualizing the N electropherograms as an overlay. Then, each electropherogram was quantitated by supplying CRT and ΔW for all peaks via the HappyTools analysis file. Peak area was calculated using the Riemann sum


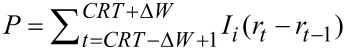


where *I_i_* is the peak intensity and rt is the migration time at *t* in the electropherogram.

For quantitation comparison, HappyTools Gaussian fitting and the Sciex 32Karat version 10.1 default peak area functionality was also calculated.

## Supporting Information

File 1 Additional tables and figures.

File 2 Variability standard deviation data.
