# Supplementary material for: Clustering and curation of electropherograms: an efficient method for analyzing large cohorts of capillary electrophoresis glycomic profiles for bioprocessing operations
Source: Beilstein J Org Chem. 2020 Aug 27;16:2087–99. doi: 10.3762/bjoc.16.176 (PMC7476600; doi:10.3762/bjoc.16.176)
Supplement: File 1 — Additional tables and figures. [file Beilstein_J_Org_Chem-16-2087-s001.pdf]

BEILSTEIN JOURNAL OF ORGANIC CHEMISTRY

Supporting Information

for

Clustering and curation of electropherograms: an efficient method for analyzing large cohorts of capillary electrophoresis glycomic profiles for bioprocessing operations

Ian Walsh, Matthew S. F. Choo, Sim Lyn Chiin, Amelia Mak, Shi Jie Tay, Pauline M. Rudd, Yang Yuansheng, Andre Choo, Ho Ying Swan and Terry Nguyen-Khuong

Beilstein J. Org. Chem. **2020,** *16,* 2087–2099. doi:10.3762/bjoc.16.176

Additional tables and figures

License and Terms: This is a supporting information file under the terms of the Creative Commons Attribution License (https://creativecommons.org/licenses/by/4.0). Please note that the reuse, redistribution and reproduction in particular requires that the authors and source are credited.
The license is subject to the *Beilstein Journal of Organic Chemistry* terms and conditions: (https://www.beilstein-journals.org/bjoc)

Supplementary information

Supplementary Table 1. Statistics on the data used throughout the work.

	**Technical replicate 1**	**Technical replicate 2**	**Technical replicate 3**	**Used for assessment**
**Biological replicates**	12 days of:<br>condition 1<br>condition 2<br>condition 3<br>condition 4<br>condition 5<br>condition 6<br>condition 7<br>condition 8<br>condition 9<br>condition 10<br>condition 11	12 days of:<br>condition 1<br>condition 2<br>condition 3<br>condition 4<br>condition 5<br>condition 6<br>condition 7<br>condition 8<br>condition 9<br>condition 10<br>condition 11	12 days of:<br>condition 1<br>condition 2<br>condition 3<br>condition 4<br>condition 5<br>condition 6<br>condition 7<br>condition 8<br>condition 9<br>condition 10<br>condition 11	(11 conditions<br>x 12 days<br>x 3 technical replicates)<br>-5 sampling errors<br>=<br>391<br>electropherograms

Supplementary Table 2. Glycans identified. SNFG is the Symbol nomenclature for graphical representations of glycans. Oxford are glycans represented using oxford nomenclature.

SNFG	Oxford	ATPS GU
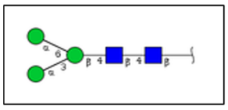	M3	4.925
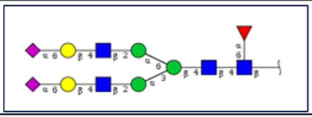	FA2G2S2	5.047
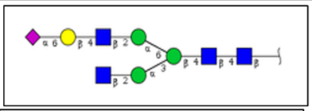	A2[6]G1S1	5.519
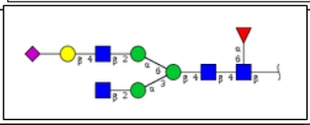	FA2(6)G1S1	5.903
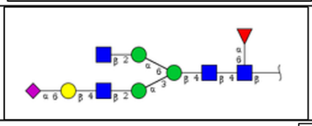	FA2(3)G1S1	6.081
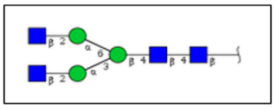 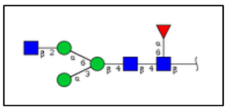 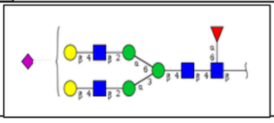	A2/FA1[6]<br>FA2G2S1	6.736/6.81<br>0/6.846
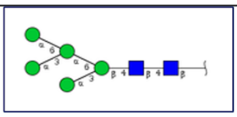	M5	6.857
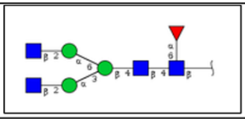 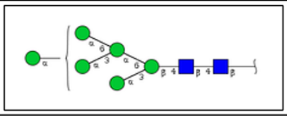	FA2/M6	7.756/7.75<br>9
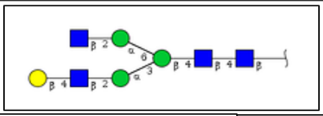	A2[3]G1	8.153
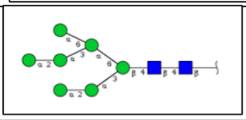	M7 D2	8.512
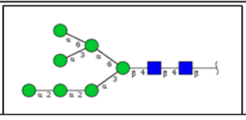	M7 D2	8.694
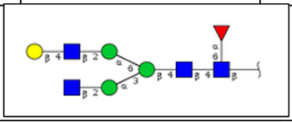	FA2[6]G1	8.838
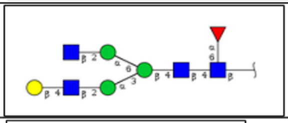	FA2[3]G1	9.184
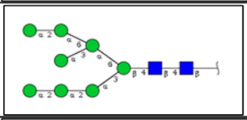	M8 D1,D3	9.693
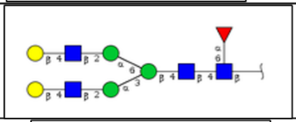	FA2G2	10.224
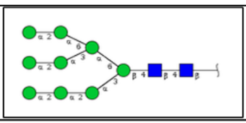	M9	10.306

Supplementary Table 3. The peak calibrated migration times (MT) and windows (ΔRT) used to define peak boundaries in all 391 calibrated electropherograms. This table was input to HappyTools via its analysis input file. Using the peak definitions in this table, peak areas were integrated separately for the 6, 30 and 355 electropherograms for cluster 1, 2 and 3 respectively.

**Cluster 1**			**Cluster 2**		**Cluster 3**	
**Peak**	**Time**	**Window**	**Time**	**Window**	**Time**	**Window**
1	2.9390	0.0230	3.0825	0.0145	2.9943	0.0205
2	3.0475	0.0165	3.1640	0.0110	3.1090	0.0180
3	3.1225	0.0185	3.2260	0.0170	3.1889	0.0330
4	3.2790	0.0160	3.3550	0.0120	3.3451	0.0150
5	3.3100	0.0130	3.3815	0.0145	3.3660	0.0140
6	3.4560	0.0330	3.5190	0.0290	3.5190	0.0300
7	3.5120	0.0160	3.5645	0.0145	3.5750	0.0150
8	3.5570	0.0160	3.6130	0.0130	3.6170	0.0190
9	3.5915	0.0115	3.6465	0.0075	3.6580	0.0100
10	3.6140	0.0110	3.6625	0.0095	3.6760	0.0110
11	3.6560	0.0320	3.6990	0.0180	3.7100	0.0200
12	3.7130	0.0200	3.7620	0.0190	3.7745	0.0185
13	3.7835	0.0125	3.8285	0.0125	3.8725	0.0135
14	3.8100	0.0130	3.8585	0.0165	3.8985	0.0145
15	3.8650	0.0160	3.9090	0.0140	3.9275	0.0145
16	3.8985	0.0135	3.9440	0.0120	3.9665	0.0195
17	3.9255	0.0125	3.9690	0.0130	4.1110	0.0170

Supplementary Table 4. Identified glycans and GU database matching statistics. Red columns were not identified in the UPLC-MS innovator (green columns identified in UPLC-MS). Average RT: raw migration time, before calibration with HappyTools, averaged over all occurrences ('#appearances' row). Supplementary Table 2 peak: the ordering of peaks and similar migration times allowed us to associate peaks in Table 1 with glycans. Average, min and max GU: the average, minimum and maximum of all of observed GU '#appearances' values. Closest DB: the closest GU value in the Sciex CE APTS database. DB GU window: tolerance of GU value matches (correspond to horizontal error bars in B). DB error: |Average GU - Closest DB|. Co-elutions are shaded the same color.

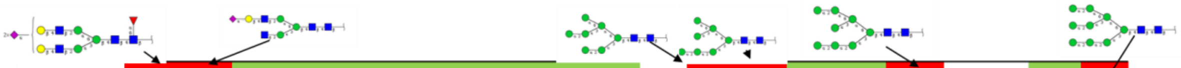

Annotation	FA2G2S2	A2[6]G1S1	FA2(6)G1S1	FA1	A2	FA2G2S1	M5	FA2	M6
Average RT	2.985	3.099	3.180	3.343	3.343	3.343	3.360	3.515	3.515
Table 1 peak	1	2	3	4	4	4	5	6	6
Average GU	4.943	5.495	5.905	6.751	6.751	6.751	6.870	7.716	7.716
Min GU	4.924	5.476	5.856	6.718	6.718	6.718	6.848	7.682	8.032
Max GU	5.012	5.677	5.944	6.790	6.790	6.790	6.898	7.752	8.113
#appearances	100	157	376	337	337	337	67	391	283
Closest DB	5.047	5.519	5.903	6.810	6.736	6.846	6.857	7.756	7.759
DB GU window	0.050	0.059	0.059	0.050	0.070	0.055	0.083	0.066	0.099
DB error	0.104	0.024	0.002	0.059	0.015	0.095	0.013	0.040	0.043

Annotation	A2(3)G1	UNK1	M7[D2]	M7[D3]	FA2(6)G1	FA2(3)G1	M8[D1D3]	UNK2	UNK3	FA2G2	M9
Average RT	3.587	3.618	3.663	3.681	3.710	3.773	3.874	3.874	3.891	3.965	3.977
Table 1 peak	7	8	9	10	11	12	13	14	15	16	17
Average GU	8.065	8.296	8.535	8.624	8.815	9.174	9.594	9.739	9.862	10.251	10.362
Min GU	8.262	8.506	8.560	8.786	9.145	9.564	9.719	9.839	10.227	10.346	10.346
Max GU	8.327	8.567	8.650	8.848	9.207	9.628	9.768	9.887	10.290	10.373	10.373
#appearances	237	279	41	391	391	128	47	116	356	26	22
Closest DB	8.153	NA	8.512	8.694	8.838	9.184	9.693	NA	NA	10.224	10.306
DB GU window	0.065	NA	0.112	0.106	0.098	0.060	0.098	NA	NA	0.095	0.088
DB error	0.088	NA	0.023	0.070	0.023	0.010	0.099	NA	NA	0.027	0.056

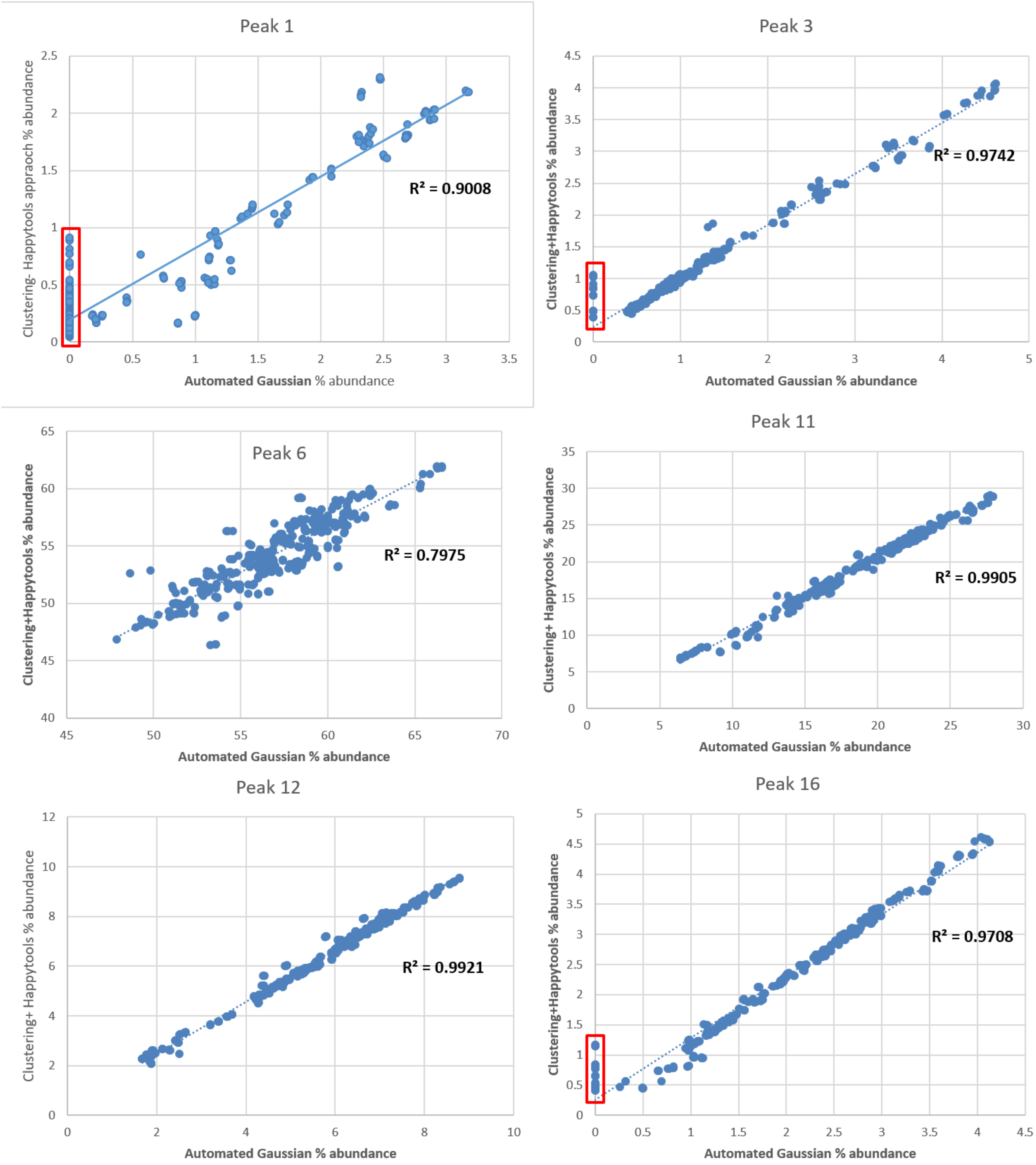

Supplementary Figure 1. Correlation of the quantitation for the automated Gaussian approach (x-axis) compared to our clustering+HappyTools quantitation (y-axis) for the same glycan peaks. Correlations only shown for the major peaks (at least one peak in the 391 electropherograms > 3%). The red rectangles show cases where the Gaussian automated approach missed peaks. The correlations for peaks 4 and 5 are shown in Figure 2 G, H and I.

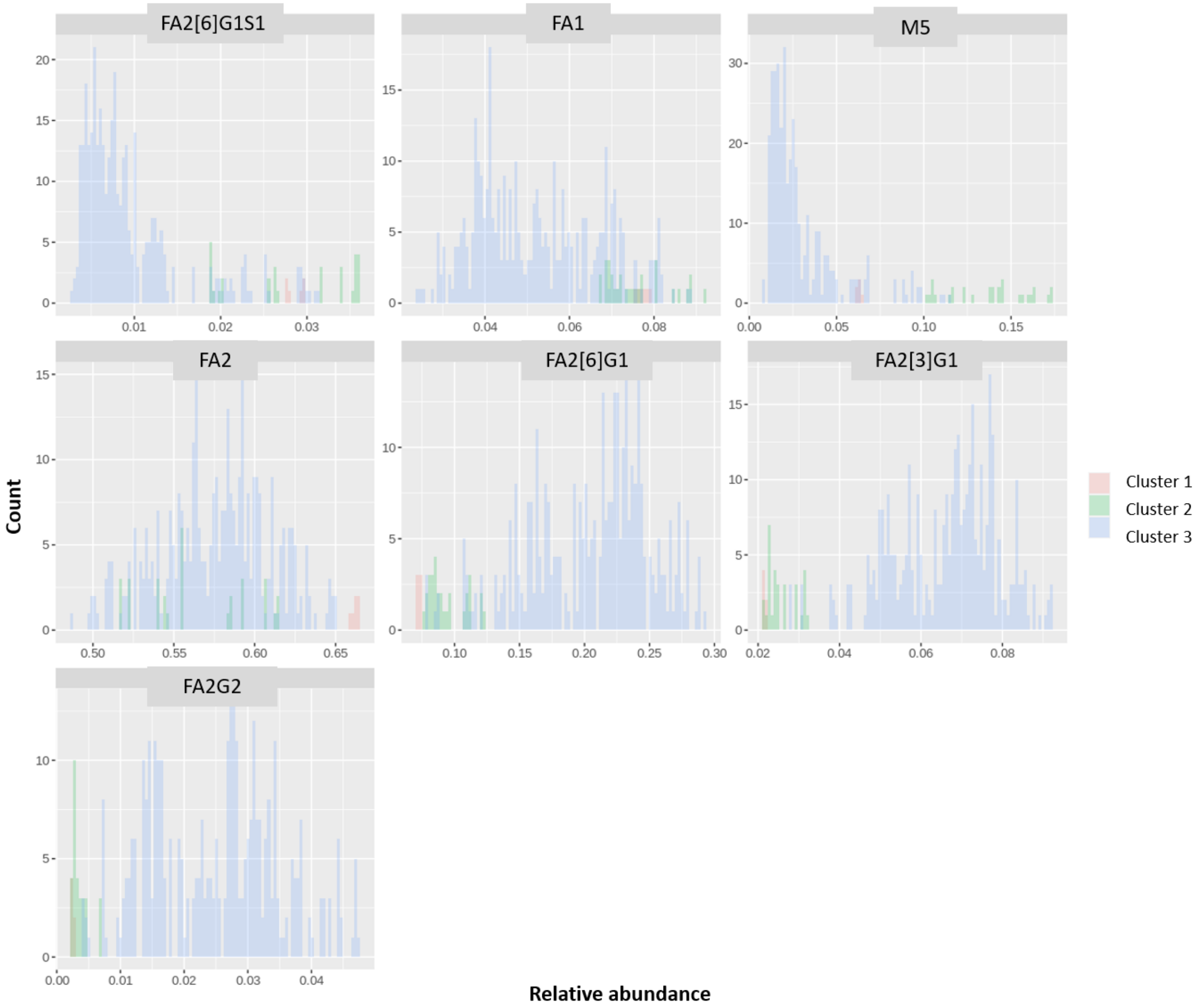

Supplementary Figure 2. Distribution of glycan abundances in the three clusters for all glycans >1% average abundance. Cluster 3 had the largest number of observations. Cluster 1 had higher abundance of FA2[6]G1S1, FA1, M5 and lower abundance of FA2[6]G1, FA2[3]G1, and FA2G2. Cluster 2 had higher abundance of FA2[6]G1S1, FA1, FA2, M5 and lower abundance of FA2[6]G1, FA2[3]G1, and FA2G2.

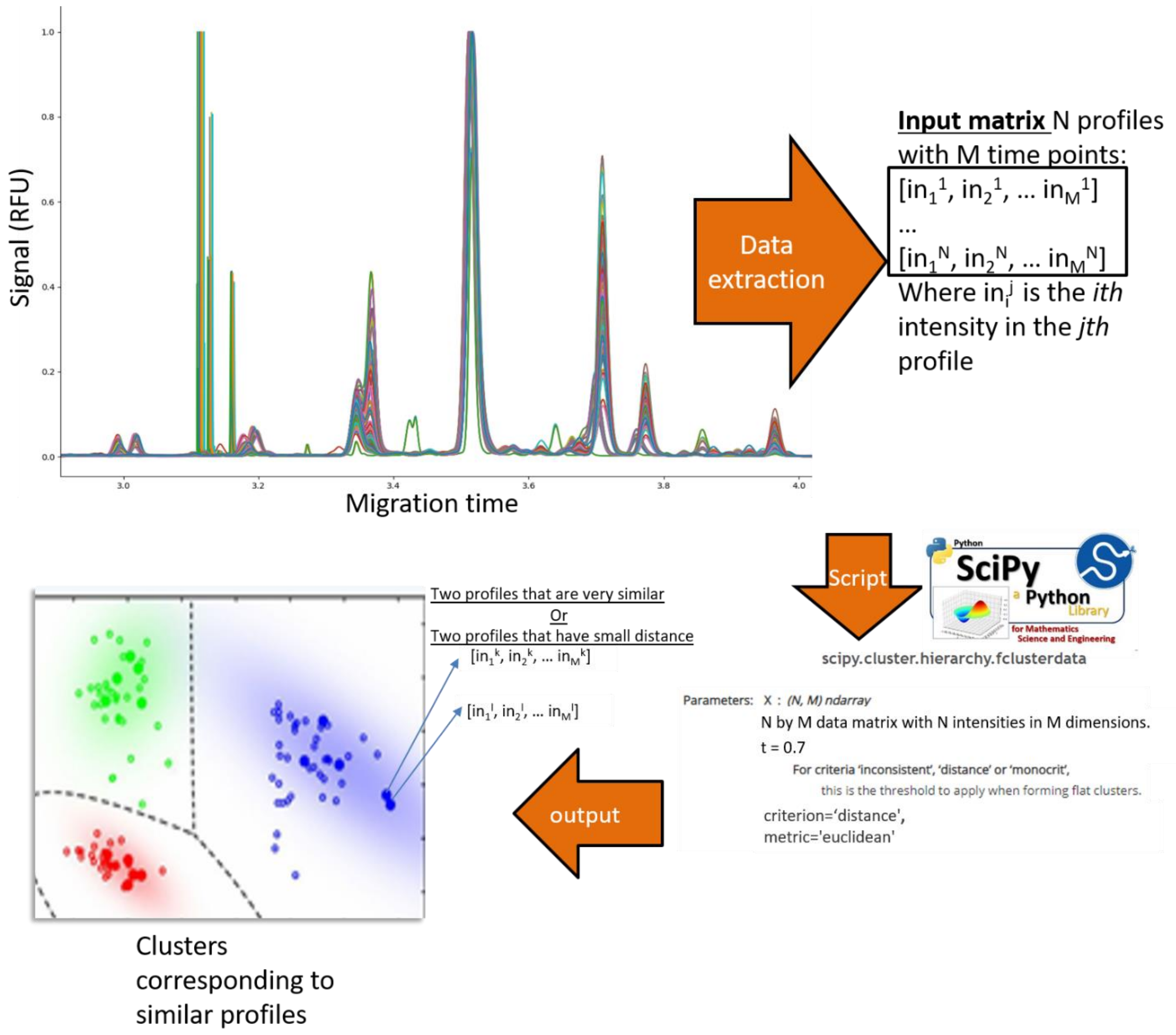

Supplementary Figure 3. The clustering algorithm in more detail. Python packages were used for clustering using the hierarchical Euclidean distance function.
